# Implementation of E-prescription for Multidose Dispensed Drugs: Qualitative Study of General Practitioners’ Experiences

**DOI:** 10.2196/27431

**Published:** 2022-01-17

**Authors:** Monika Knudsen Gullslett, Trine Strand Bergmo

**Affiliations:** 1 Norwegian Centre for E-Health Research University Hospital of North Norway Tromsø Norway

**Keywords:** e-prescribing of multidose drug dispensing (eMDD), pharmacy, start-up, general practitioner (GP), Norway, digital health, digital tools, e-prescriptions, physicians, qualitative study

## Abstract

**Background:**

Increased use of pharmaceuticals challenges both capacity and safety related to medication management for patients and changes in how general practitioners (GPs) and other health personnel interact with and follow up with patients. E-prescribing of multidose drug dispensing (eMDD) is 1 of the national measures being tested in Norway.

**Objective:**

The objective of this study is to explore GPs’ experiences with the challenges and benefits of implementing eMDD in Norway.

**Methods:**

Qualitative in-depth and group interviews were conducted with a total of 25 GPs between 2018 and 2020. Transcribed files were saved in NVivo to conduct a step-by-step content analysis. NVivo is a software tool for organizing, managing, and analyzing qualitative data.

**Results:**

The study revealed that eMDD offers many benefits. At the same time, there are several challenges related to information, training, and initiation, as well as to the responsibility for the medication, interactions, and the risk of incorrect medication. An important activity in the start-up phase was an information meeting with pharmacies and technology suppliers, as well as exchanging information and instructions with pharmacies on how to get started. Four analytic themes emerged through the extraction of data: (1) start-up with eMDD (“Be patient”); (2) the need for training; (3) interaction, safety, and efficiency; and (4) the working day with eMDD.

**Conclusions:**

There is a variation in different GPs’ needs regarding training and information, and considerable variation in competence and motivation related to the use of digital tools. There are also different degrees of understanding the everyday work of the other actors in the medication chain. In particular, the harmonization of medication lists related to the use of time, expenditures, and challenges with technological solutions in the introduction phase was emphasized as a challenge. Overall, GPs who have started using the system report great benefits; these are largely related to an increased overview of patients’ total medication lists, less time spent on prescribing prescriptions, and increased collaboration with pharmacies and nurses, both in service from providers in homes and in nursing homes.

## Introduction

### Background

Digitalization and the use of electronic systems to manage medication are salient elements in developing future health care services that have a current political, clinical, and research focus [1,2]. With a population characterized by an increasing proportion of fragile and older people and people in need of health care, the use of different types of medication is also increasing [3-5]. Changes in population structures have also resulted in a need to change how general practitioners (GPs) and other health personnel perform their tasks and how patients are medicated [6]. This also applies to the handling of medications for people who have several diagnoses (multimorbidity) and who are thus dependent on several types of medication (polypharmacy) [7-9]. In Norway, among other countries, a recent change has been the introduction of e-prescribing of multidose drug dispensing (eMDD) [10-12]. The goal of e-prescription technology is to contribute to a more conscious and safer use of multiple medications by a single individual [13,14]. It is expected that technology, such as the use of eMDD and other solutions, will reduce duplication of medications, contribute to correct dosages, and reduce confusion among providers and patients [12,15].

### What Is MDD, and What Is eMDD?

MDD was introduced in the early 2000s to reduce errors and streamline the distribution of medications in municipal health services [6,8]. The main goal of introducing MDD was to reduce incorrect dispensing, save time for health providers, and reduce the disposal of medications [11,16,17]. MDD is intended to replace pill dispensers and is a mechanical system used by central pharmacies to package medications in small unit-of-use bags, with 1 bag for each dosing time. Users receive a strip with many small bags marked with the patient ID, medication information, and time of ingestion. MDD is packaged and delivered every fortnight. Today, more than 90,000 people use MDD in Norway [18]. Of these, 68,400 (76%) receive service from professional caregivers, 18,900 (21%) live in nursing homes, and just under 3600 (4%) receive multidose drugs by private agreement with a pharmacy [1]. GPs prescribe multidose drugs by listing the patient’s medications on a prescription card. This medication list is then printed out and mailed or faxed to the pharmacy. Once a GP signs this medication list, it is valid as a prescription for 1 year.

Errors in the e-prescribing of medication as incorrect medication may be a serious problem [8,19]. Several issues, such as training staff, designing routines, and focusing on the environmental aspects of the practice, are important for avoiding such errors [19]. In Norway, safe digital routines are increasingly being designed and implemented to prevent incorrect medications, improve patient quality of life and safety, and contribute to a more efficient workday for practitioners, including GPs [7]. As part of the digitalization of health services, Norwegian health authorities have begun testing eMDD within the e-prescription solution [20]. In this paper, we focus on the challenges and benefits GPs experience when implementing eMDD.

Today, over 90% of prescriptions are sent to the pharmacy as e-prescriptions [21]. Among the medications still prescribed on paper are multidose drugs; in Norway, the goal is to transfer these to the e-prescription system. Toward this end, the Norwegian health authorities have begun testing eMDD within the e-prescribing system. eMDD means that an electronic medication list and e-prescriptions replace the paper list and fax. The GP sends a list of the patient’s regular medications to the prescription database, together with e-prescriptions, as the medication list has no prescription function [22]. The prescription database is a central database where prescribing information is shared between all health personnel with prescribing privileges and all pharmacies. In the eMDD system used by the GPs in our study, an electronic MDD message that includes the medication lists was added to the e-prescriptions. The medication list for MDD is called the “medications in use” list (or just the patient’s medication list). The patient’s medication list shows the patient’s regular medications, medications as needed, dietary supplements, and any critical information related to the medications, as well as recently discontinued medications. To be able to submit an MDD notification to the prescription database, the doctor must first register as an MDD-responsible doctor. The medication lists and e-prescriptions are developed in the GP’s prescribing module in the medical record system and sent to the prescription database. The pharmacy can access the information and transfer it to the packing machine for dispensing. Prescriptions are displayed in the prescription database 1 month after they have expired. In the e-prescription system, the responsible doctor and the pharmacy electronically communicate regarding any necessary clarifications. The home care staff receives an e-message notification when GPs make changes to patients’ medication lists [19]. The target group for eMDD in Norway is currently patients in municipal nursing and care services. There is some variation in the user groups related to age and, among other things, the use of medications.

Going from paper to electronic medication lists also makes medication information available in the prescription database to medical doctors in emergency rooms and hospitals, making medication information more available during care transitions. Moreover, the GP receives a notification from the pharmacy if there are changes in the medication treatment that have not been initiated by the GP [1].

Studies have highlighted a significant decrease in the number of discrepancies between the medication lists at GPs and pharmacies when eMDD is compared with MDD prescribed using paper and fax [22]. In addition, research related to digitalizing prescriptions has found that health professionals experience the solution as a quality improvement [23]. Results from another study focused on the potential to streamline workflow for health care providers and minimize interruptions from, among other things, the use of phone and fax communications. This study also emphasized that technical standards and system design changes, and more targeted training, may be needed to address barriers to e-prescriptions [24].

### Context for This Study

The goal of the digital solution being implemented is to provide a safer system based on electronic routines and updated medication lists. As stated, health authorities expect eMDD to reduce incorrect use of drugs, but research in the field is sparse, and studies from other countries cannot be transferred directly to the Norwegian context due to other systems and routines for eMDD [17]; this study also found that health professionals experience the solution as a quality improvement [17]. Discrepancies in the medicine list are also a challenge in transition between institutions, or institutions and homes, in the processes of admission and discharge from the hospital, and electronic tools may be helpful to avoid or minimize medication discrepancies [25].

eMDD was piloted in 24 GP clinics/offices in the southern part of Norway between 2018 and 2020. As part of this pilot, we undertook a study investigating how the GPs experienced the transition from prescribing MDD using paper and fax to eMDD. This paper presents findings from interviews conducted with GPs in different parts of the country based on the research question, *What are GPs’ experiences with their challenges and benefits regarding the introduction, implementation, and use of eMDD in their practices?*

## Methods

### Research Design

A qualitative, explorative study using in-depth interviews was conducted in GP practice. The study’s methodological approach was based in the social sciences, using an abductive strategy that aimed to uncover—and then interpret—knowledge about the social actors in question [26]. Different research strategies are summarized by Peirce [27]: “Deduction proves that something must be; induction shows that something actually is operative; abduction merely suggests that something may be.” The abductive strategy works well with the hermeneutic-phenomenological approach we used in the analysis; moreover, the choice of research strategy was integrated into the study's objectives and the research questions under investigation. In this study, the choice of a hermeneutic-phenomenological perspective means that the researchers tried to achieve an in-depth understanding of the study participants’ life-world experiences with the topic of the study and to uncover and interpret knowledge about GPs’ experiences with implementing eMDD [28,29]. Even if the data gathering and analysis are done with a reflexive and open-minded view, the researcher’s hermeneutic position will affect the results based on the theoretical approach and their preconceptions [30,31].

### Selection, Sample, and Interviews

The findings in this paper build on this knowledge and focus on the experiences GPs have related to the implementation and use of eMDD. Experiences from the pilot eMDD and whether the system meets expectations were investigated. The Results section emphasizes the GPs’ work situation and patient safety—that the patient receives the right medicine at the right time—and the analysis places the eMDD experiences in light of the complexity it is part of and also ensures patient safety and the correct use of medicines for patients who are prescribed medicines through eMDD. All GPs who implemented eMDD between 2018 and 2019 were invited to participate in the study. A total of 24 GP clinics/offices were involved in testing eMDD during this period. We received contact information for these offices from the Directorate for e-Health in Norway. A total of 26 GPs from 10 doctors’ offices agreed to participate, of which 3 (11%) agreed to participate in a follow-up interview. A qualitative, explorative study using in-depth interviews was conducted by both authors to investigate how GPs experienced the introduction of eMDD. A third researcher (EJ) conducted interviews together with researcher TSB.

Two focus groups were conducted, with 8 doctors in each interview and 9 individual interviews, 4 of which were telephone interviews. In addition, 1 GP described experiences with eMDD in 2 e-mails. The GPs had between 5 and 20 patients who used MDD. Some GPs were salaried, while others worked on a contractual basis. Some were interviewed after 2-4 months of use and others after 1 and 2 years of eMDD use; 3 GPs were interviewed again after 10 months of use (these were from GP clinic 6 in Table 1). Both women and men participated in the individual and focus group interviews. The interviews lasted from half an hour to three-quarters of an hour, depending on the informants' information and schedules. The interview locations were conducted either in the GPs’ offices or digitally via Skype for Business. Skype for Business was a solution to complete the data gathering after the lockdown restrictions that started in March 2020 due to COVID-19. Before each interview, the authors informed the participants about the project, and the GPs provided both written and verbal consent to participate in the study. The interviews were based on a semistructured interview guide, developed to obtain knowledge about issues related to the implementation and use of new technologies (eMDD). All the interviews began with an open question concerning the informant’s experiences with eMDD. To ensure that the research question was covered, the interview guide was used as a checklist during the interviews [30]. In addition to the included questions in the guide, topics raised during the interviews were followed up, when appropriate, to obtain in-depth knowledge related to important issues for the GPs. The main topics in the interview guides concerned:

- How the doctors experienced using eMDD
- How the doctors experienced the start-up phase
- What changes occurred in the GPs’ organization of clinical work
- What the doctors experienced as positive and as negative
- What the doctors felt could be improved

**Table 1:** Information about participants (N=26) and interview details.

GP^a^ clinic number	Participants	GPs interviewed, n (%)	Interviews	Setting	Researcher
1	County 1	8 (30.8)	Focus group f2f^b^	Urban	TSB/EJ
2	County 2	1 (3.8)	Telephone interview	Rural	EJ
3	County 2	1 (3.8)	Telephone interview	Rural	EJ
4	County 2	1 (3.8)	Telephone interview	Rural	EJ
5	County 2	1 (3.8)	E-mail interview	Rural	EJ
6	County 3	8 (30.8)	Focus group f2f	Urban	TSB/EJ
7	County 3	1 (3.8)	Telephone interview	Urban	MKG
8	County 4	3 (11.5)	Individual interview f2f	Urban	MKG
9	County 4	1 (3.8)	Individual interview f2f	Urban	MKG
10	County 4	1 (3.8)	Individual interview f2f	Rural	MKG

^a^GP: general practitioner.

^b^f2f: face-to-face communication/meeting.

### Analysis

The in-depth interviews were digitally recorded and then transcribed verbatim by a professional company. All the transcribed interviews were saved as files in NVivo (QSR International) to systemize the analysis [32]. Both authors were responsible for the interviews and the analysis of the material and also thoroughly discussed this several times during the analysis process. Both authors read all the interviews. To analyze the data, 4 steps of systematic text condensation were followed [30,33]. The authors first read all the interviews, initially to obtain a general impression and then to identify key themes. The authors read the interviews with special attention to the GPs’ experiences during the start-up phase. NVivo was used to systematize relevant text [34] and then discussed and agreed on the key themes, categorized the text, and adjusted it, as needed. The categories were developed through an abductive and iterative process based on the topics in the interview guide [26]. The text was then condensed, analyzed, and discussed further, and finally merged into the revealed themes. The key themes are presented in the Results section, augmented by illustrative quotes.

We used a professional agency to translate from Norwegian to English.

### Ethical Assessment

The project was approved by the data protection office (DPO) at the University Hospital of North-Norway (UNN; ethical approval no. 02003). The GPs involved in the study received both written and oral information about the study and were guaranteed anonymity before they agreed to voluntary participation. Information was given explaining that they could withdraw from the study at any time. The data are anonymized in the presentations.

## Results

### GPs’ Experiences and Description of Using eMDD

In this section, the GPs’ experiences and description of using eMDD are illustrated by presenting the findings, addressing the following research question: *How do GPs experience the introduction and use of e-prescribing for MDD?* The findings are represented by 4 emergent analytic themes: (1) start-up with eMDD (“Be patient”; (2) the need for training; (3) interaction, safety, and efficiency; and (4) the working day with eMDD.

#### Start-up With eMDD: “Be Patient”

Prior to the eMDD start-up, a joint introductory meeting was planned and held with each GP’s office, with a video conference with pharmacies, the Norwegian Directorate of Health’s IT department, and the GPs and technology suppliers. The objective of this meeting was to review how the introduction of eMDD was to be carried out technically and how the lists were to be submitted. The GPs described this meeting as useful, as was the manual sent by the pharmacy with a description of how the MDD patients should initially be registered. Registration was experienced as the most demanding process, and GPs responsible for several MDD patients reported a considerable uptick in work during the registration process. Shortly after the doctors signed up for the system as the responsible MDD GPs, they could start registering patients and medication lists. Several of the GPs emphasized the importance of having patience during the start-up process.

[You must] be patient with it. And be prepared that you may get a lot of messages from the pharmacy in the beginning. In the very stressful everyday life of general medicine, it can seem a bit like an extra stress in the beginning. But eventually, it becomes just part of the job, and then it becomes much easier. You also get a lot back and forth with the home service regarding medication. Be patient at first.

Some GPs also experienced a number of technical problems when they had to register patients. The technology was slow at start-up, and there was a considerable delay between when the information was added and when it was processed into the database. The GPs found it difficult to get started and described it as a slow system. This was revealed as a major obstacle in their everyday work, as it was not possible to do other tasks on the computers while the system was working on storing information. As a result, a number of GPs opted to spend time on this registration process in the evenings and on weekends.

The GPs’ overall experience was that the registration took approximately 20-30 minutes per patient or MDD, reconciling the lists that were in different places (ie, at the pharmacy and with the doctor) and registering responsibility for the medication. Here, ensuring the coordination of lists in advance of the start-up proved to be an advantage. Lists from the pharmacy had been sent together with information about how to start with eMDD a few weeks before, which made it easier to complete the registration.

We first got a list from the pharmacies to know who our MDD patients were, and then we started the process of cleaning up medication lists and preparing. It was really like a pre-release, that. So, it was a long time in advance, so we had the opportunity to start working on it. But it still took a long time. However, you must go through each registration to see if it is correct. So, this took time anyway, even though all the work had been done in advance.

According to some GPs, although there were only a few patients per doctor, they still experienced the job of converting to MDD e-prescriptions as an inconvenient but necessary task.

It is a bit of a job but not something that is unmanageable. What we have to think about is how the information should be provided, because since we have a pilot, we had meetings for this where we actually get paid to show up. That will not happen when this is rolled out, broadly.

It was pointed out that the GPs who have the most patients on MDD will have a tremendous workload in the introductory phase but will probably also be the ones who get the most out of it once the system is up and running. One issue related to the introduction of eMDD was the uncertainty regarding the number of resources required to get started. As the GPs already felt overloaded with a considerable number of tasks in their everyday work life, it was a source of concern that there could be additional workload with a new system. To help achieve a smooth introduction, the GPs suggested that the workload associated with the introduction be made visible.

I think it’s very good that when you start a project, it’s just the beginning of a change that is for the better, and it’s good to know what it entails. I would appreciate the project manager being honest and open and saying something about what to expect when we join.

A clear expectation that emerged from the interviews was that eMDD would contribute to a simpler everyday work life for GPs and increase the quality and safety concerning the handling of medication. Several GPs emphasized that the benefit of such a system is safety: multidose drug prescriptions on paper and the use of fax for communication between different parties was described as a low-functional and old-fashioned system. They noted that the use of multidose drugs was often cumbersome and time-consuming and that it could be difficult to keep track of medications. As such, expectations for improvement were high.

So, a multidose is sometimes terrible, like that in paper form with lots of sources of error and lots of nonsense, faxes, and forms and triple lists and all that, so I ended up having to say no to MDD for new patients, because that scheme was low-grade quality. Getting an electronic multidose [system] has been welcome and something we have been waiting for.

The motivation for several of the GPs was that any time spent getting started with the new system was something they would get back later.

#### The Need for Training

The GPs had different experiences concerning training to use the new system. Some GPs found that a letter from the pharmacy was sufficient for the start-up process, while others would have preferred to have attended courses. Those who were satisfied with the training using eMDD reported that having specific people (ie, at the pharmacy or technology supplier) to contact when they needed help with something was important. It was also easier for the GPs to become acquainted with getting started in doctor’s offices where several others were interested in and had familiarized themselves with eMDD.

It was perceived as a problem by the GPs if there was no access to people who could be available as a resource (ie, those with a deeper understanding of the system). If a resource person was not available, this had a negative effect on implementation. Here, tasking someone with the role of a “superuser,” who would get to know everything thoroughly and could be available as a resource for others, was recommended. Moreover, several of the GPs expressed that they had received neither the necessary information nor training and wished that someone had come to the office to introduce the project. A day-long conference with information and training with the project leaders was also recommended.

I wish they were clearer on the information about when it was to begin, and preferably, well, there are quite a few of us who are not too good at data (technology use), so to at least consider whether one can collect or create . . .

The GPs pointed out that those who practiced in offices with fewer employees may have greater problems getting acquainted with new systems if they do not have a large patient base to register in the system. The GPs also pointed to training-related challenges as being rooted in a mixture of pedagogical shortcomings and some technical issues that made the initial workload heavier than it should have been. Here, the need for simple, quality training was highlighted—particularly training that could be undertaken during the workday rather than during the doctors’ free time. In the interviews, it emerged that the GPs wanted to learn more about the system: for example, what the pharmacy sees on the screen when the doctor sends something and what the hospital doctor can and should do with the medications being taken (ie, the patient’s medication list). The GPs emphasized the necessity of adapting training related to their different needs, especially since there was variation in both their interest in and their desire for the digitalization of MDD. With regard to training, video clips and help from colleagues were highlighted as useful.

#### Interactions, Safety, and Efficiency

One of the most important tasks when first implementing eMDD is to clean up and update the medication lists with the correct information, and all the GPs described this as a tremendous undertaking. They also explained that it was important to approve the lists, be clear on how dosing takes place, and ensure that this is stated clearly; however, they experienced this as challenging when the system did not work properly.

It was a bit chaotic. We thought we had to delete old papers, and for some patients, there were huge lists of old prescriptions saved. But then we found out that it was possible to update without deleting old papers, and that made everything a little easier.

The GPs described this process as quite labor intensive and, for many, surprising. Several related that they had not been mentally prepared for so much work, even though they had been informed well in advance to update the medication lists. One GP explained:

We had been sent what the pharmacy and the home service had on their lists, so it was up to date, and we thought it was mostly a push of a button. I’m not that computer savvy, and it took a lot of time. It was the use of time that was the problem [. . .] So, there was a lot of work then to start the process of getting an overview of all the lists.

Another one shared:

Yes, so the advice is that you must always have an overview of your patients’ medications and that you must enter and clean up the medication lists continuously. It must be “up to date.”

GPs described having to spend time cleaning up after hospital doctors who had prescribed new medications without deleting the valid prescriptions that were already in place, as the official regulations state that this is the GPs’ responsibility. They, therefore, recommended that there must be an implementation period in which time and resources are set aside for training so that everyone understands the importance of doing this.

GPs described both positive and negative experiences related to interaction and safety when using eMDD. For example, the nursing and care e-messages between GPs and the home care staff were experienced as smart and were defined as a “safety valve” concerning communicating changes in the medication lists. The GPs felt this provided a better overview for all actors with regard to determining the correct medication. However, communicating changes in the medication list to the multidose pharmacy was experienced as more uncertain:

I’m not always quite sure if they got it. It has been—or we have to write physically as a message at the bottom, “I have changed so and so.” I have actually experienced that they have not done exactly as I have said.

This challenge was explained as being partly due to a lack of knowledge regarding what the technical aspect looks like at the pharmacy (ie, whether it is physically possible for the GP to make a mistake when sending an MDD list to them). The question is whether it goes to a machine and the machine makes all the mistakes or whether it is the case that a person is responsible for what is to be in each small bag.

Several points emerged in the interviews related to weaknesses in the safety of medication use for patients. A problem highlighted by all involved parts in the medication chain was that there was a big safety gap related to the fact that medications prescribed with e-prescriptions can be picked up twice. The GPs pointed to an example: if an electronic prescription is legal for a year, the system is not structured so that it is locked in the multidose drug list. This means that the patient can pick up the medication by themselves, regardless of what is packaged in the MDD. The pharmacy should be able to determine that the prescriptions have all been picked up, but instead, they are packaged in the MDD, and the user will get double medication. To increase safety and overview for all parties, the GPs thus stated that it is important to emphasize a thorough review of medication lists, structured as part of their everyday workday.

The important thing was to have updated medication lists, that we had to make sure that we did not have any magistral prescriptions, or any reminders we had to ourselves, or that there were messages to the home care staff in the medication lists. Because there were some things we did before to make things work, which do not work at all if you have e-multidose. So, it took a while to clean up those lists.

#### The Working Day With eMDD

The GPs also had different experiences around the use of eMDD in their everyday workday—this seemed related to whether there were clear lines of communication between all involved parties. One frustration noted by many of the GPs was that changes made to the system were not always registered. They would then receive a message from the pharmacy to discontinue and recall the medication list and prescribe again. One GP shared this experience:

Sometimes, I have tried to discontinue medication 5 times and yet it has not worked. Then the message comes back from the pharmacy, and they write smiley faces and try to be nice to us, and say we are sorry, but you actually have to stop again and prescribe again.

One GP suggested a phone-a-friend approach as a solution to this issue.

There are programs on TV that have an option called “phone a friend.” And you can at least do that at least once, so I can tell you that you are allowed to call me in the evening, but sitting together and watching it together, I think that might have solved his frustration and your problem, so probably everyone would have saved time.

Many GPs reported that after the initial start-up process, once the system had been in use for a while, it facilitated a better working day. As one stated,

Errors in the lists—they are not there anymore. So now there is a good flow in our workflow, so it is an integral part of our everyday life that we do not think so much about anymore.

Another GP shared his experience:

I am very happy with e-multidose, it is very good. We can reduce the use of paper—as long as it works, it is absolutely fantastic. So, it’s just to make it work, but lately it has been very smooth, so there have been no problems in recent weeks, and very few messages from the home nurse and from the pharmacy. When things are established, it rolls smoothly.

Even if it was a challenge during the implementation phase, most of the GPs welcomed the eMDD:

I think no one really knew what they were getting into, so everyone was optimistic and looking forward to finally dropping the fax and stuff.

## Discussion

### Principal Findings

In the Results section, we presented GPs’ experiences with implementing eMDD, focusing on GPs’ information and training needs and their experiences with the start-up process, including the coordination of lists, safety and effectiveness, and changes to their working day [23]. There are variations in different health providers’ and GPs’ needs regarding training and information and considerable variation in competence and motivation related to the use of digital tools [35]. There were also different degrees of understanding concerning the everyday work of the other actors in the medication chain. In particular, the harmonization of medication lists related to the use of time, expenditures, and challenges with technological solutions in the introduction phase was emphasized as a challenge [36,37]. Overall, GPs who have started using the system report great benefits; these are largely related to an increased overview of patients’ total medication lists, less time spent on prescribing prescriptions, and increased collaboration with pharmacies and nurses, both in service from providers in homes and in nursing homes.

Previous studies have shown that better availability of patients’ overall medication increases patient safety and increases collaboration between different health care providers. In addition, access to a patient’s medication list and health information enhances safety and saves GPs time [38]. One reason is the faster updating of prescriptions electronically. One of the most positive things about eMDD from the GPs’ perspective, compared to the use of paper and fax, is a better overview of lists and that prescribing can be done immediately and increases the chances of the information arriving [22]. As such, to achieve quality implementation, it is important to develop systems that ensure quality information and training provision at start-up; it is equally important to have quality guidelines in place and technology that promotes interaction between all involved parties and ensures safety for patients [39]. To obtain this, it is important to gain a complete and accurate overview of each patient’s medication needs. It is essential that the type of medication and dosage be included in the medication list—this, in turn, ensures professional justification and enhances both the quality of services and the patient’s quality of life.

As revealed in the Results section, however, there are still several challenges associated with this. Regarding organization and collaboration, the GPs reported a lack of knowledge about what the medication chain looks like for each individual involved—a source of concern as they felt this could affect both safety and effectiveness with regard to medication management. Another challenge was the delay experienced between when information was added to the patients’ medication list and when the system reflected the updates. There are several possible solutions to this issue. This could be an opportunity for hospital doctors to discontinue a medication that should be removed from the MDD list. Increased communication and understanding of deadlines between the various actors, and a good support service related to the digital system(s), such as the technology provider, may ensure right medication. Digitalization helps ensure faster and more secure transfer of information when the technology is implemented in an appropriate way [40]. Research has shown that both GPs and employees in the home care service experience MDD as contributing to quality improvement related to patient overview and safety when patients are taking multiple medications [21]. In this study, the primary attitudes toward eMDD among the GPs were positive; they felt it facilitated better patient safety and was efficient and professionally justified. However, they also emphasized that to create and implement a well-functioning eMDD solution, collaboration between all actors is required. The question remains whether the use of eMDD actually contributes to the realization of gains via increased efficiency (ie, through reducing time spent on prescribing and improving interaction and patient safety). The process of getting started with eMDD was labor intensive for the GPs. However, once they spent the time necessary to establish an updated and correct medication list for each MDD patient, it proved a time saver during their everyday workday and contributed to increased patient safety [36]. Nevertheless, there will always be variations within and between municipalities, GPs, and the specialist service; as such, using eMDD on a broader scale in Norway and other countries will necessitate a focus on ensuring that the digital solutions are implemented with quality information, training and structure, and standardized solutions in place as far as possible.

### Implications for Practice

There are some important issues to follow up on in the introductory phase and the scaling-up process of introducing eMDD in GPs’ offices. Several of the GPs in this study looked forward to the project's start-up, yet many pointed out that it might be difficult to handle the extra tasks. To sum up, good routines are necessary for training all stakeholders, including GPs, pharmacies, municipal health services, technology suppliers, and patients, where appropriate. Making the appointment of a superuser responsible for eMDD, who can follow up when needed, is also of high importance. It was especially pointed out that a specific contact person for both GPs and nurses at pharmacies when complications occur will ease the implementation [21]. The GPs felt that increased contact and collaboration with the pharmacy could have helped simplify the work. Clear placement of responsibility for solving challenges that arise is also needed; this also applies to support for technology challenges. There must be a provision of extra time to register everything correctly when starting eMDD. Having to do the same task repeatedly was time-consuming and was noted as potentially hindering the GPs’ ability—and willingness—to implement eMDD as part of their everyday work lives. eMDD seems like a safer and more effective solution when implemented in the organization for all the included parts. Nevertheless, these data may contribute to a greater reflection on—and discussion about—the current, rapid implementation of electronic prescription of medication in the health services and the challenges that may appear.

### Limitations/Weaknesses of the Study and Issues for Further Research

The study was performed during the implementation of eMDD and followed up with a few interviews after 3-6 months to explore experiences with the start-up process of eMDD by the GPs. A potential weakness of the study is its reliance on both physical and digital interviews with GPs. As such, the information derived from the interviews may have been different if the interviews had been conducted in person. Another weakness is related to the use of different interview strategies; however, this may also have strengthened the analysis by investigating both individual opinions and opinions reflected in a group of GPs. We further acknowledge the variation of the GPs’ experience with eMDD, with some of them being experienced only for 2 months and others for 2 years, as a limitation.

### Conclusion

The literature on the topic is growing but still limited, and more research is needed to develop digital prescription of medication to enhance safety for all included parts, especially the users. Awareness of the hindrances revealed both in earlier research and in this study may strengthen the motivation and establish routines including stakeholders and support from both the pharmacies and the technology provider for launching the digital solution eMDD as a working tool for GPs. There is a need for further investigation, including qualitative research, to build solid and evidence-based knowledge that can contribute to developing tailored handling of medication for multidose drug users. Further research should focus on service users’ experiences, cocreation between different stakeholders, and how to scale up the use of eMDD, while ensuring that the use of eMDD is appropriate, safe, and available for end users (patient), next of kin, and health service providers (eg, GPs, pharmacists, and nurses).

## Abbreviations

eMDD: E-prescribing of multidose drug dispensing
GP: general practitioner
